# Predictive factors of responsiveness to a body weight reduction program in Prader–Willi patients at 6 years of follow-up

**DOI:** 10.1038/s41598-022-09096-x

**Published:** 2022-03-25

**Authors:** Stefano Lazzer, Filippo Vaccari, Mattia D’Alleva, Giorgio Bedogni, Diana Caroli, Graziano Grugni, Alessandro Sartorio

**Affiliations:** 1 Department of Medicine, University of Udine, P.le Kolbe 4, 33100 Udine, Italy; 2 School of Sport Sciences, University of Udine, 33100 Udine, Italy; 3 Department of Medical and Surgical Sciences, Alma Mater Studiorum University of Bologna, 40138 Bologna, Italy; 4 Experimental Laboratory for Auxo-Endocrinological Research, Istituto Auxologico Italiano, IRCCS, 28824 Piancavallo, VB Italy; 5 Division of Auxology, Istituto Auxologico Italiano, IRCCS, 28824 Piancavallo, VB Italy

**Keywords:** Physiology, Endocrinology, Health care, Medical research

## Abstract

Prader–Willi syndrome (PWS), a multisystemic disorder caused by lack of expression of genes on the paternally inherited chromosome 15q11.2-q13 region, is characterized by hyperphagia and childhood-onset morbid obesity, A retrospective cohort study of 60 PWS patients, 38 females and 22 males, undergoing a 6-year rehabilitation program was analysed. Mean age at the time of first admission was 27 ± 7 years, body weight (BW) was 97 kg ± 29 kg and height was 1.53 ± 0.09 m. Twenty-four patients (40%) showed BW loss after 6 years of follow-up, seventeen (28%) remained stable and nineteen (32%) gained BW. Responsiveness in term of BW reduction was less frequent in patients with the UPD karyotype, karyotype del15 being more frequent among responsive patients. Furthermore, responsive PWS subjects had a higher BMI (47 vs. 36 kg/m^2^), waist (123 vs. 106 cm) and hip (136 vs. 118 cm) circumferences than non-responsive at the time of first hospitalization. Baseline body composition and metabolic parameters did not differentiate between responsive and non-responsive patients. Given the rarity of PWS and relative lack of studies, these results can be considered relevant because based on a relatively large number of PWS patients followed up for a long term period.

## Introduction

Prader–Willi syndrome (PWS) is a rare genetic disorder with an incidence of approximately 1 in 21,000 newborns^1^, affecting both sexes with similar prevalence. PWS is the most common form of syndromic obesity and is characterized by endocrine, physical, behavioural, and intellectual abnormalities^2^.

PWS is mainly due to two different genetic mechanisms: paternal deletion of chromosome 15q11-q13 region (del15), which is the most frequent, and maternal disomy 15 (UPD) in which both chromosome 15 s are inherited from the mother. Furthermore, there are other less frequent individuals, who have defects of the imprinting centre, chromosome 15 translocations or inversions^3^.

PWS patients are characterized by early poor feeding and lack of appetite, followed by uncontrolled appetite and lack of satiety that cause obesity, mostly after the age of 2–3 years^4^. Unfortunately, these individuals have a reduced life expectancy and evidence suggests that obesity and obesity-related issues are prevalent in the deceased PWS population^5^. Besides obesity, PWS patients have lower muscles mass compared to obese subjects with similar characteristics but without PWS^6,7^. This leads to a significant reduction of energy expenditure in individuals with PWS due to low resting energy expenditure but also from reduced physical activity^8,9^.

Therefore, a multidisciplinary approach including food restriction**,** physical rehabilitation, and psychological counselling is essential in PWS patients in order to improve body composition and quality of life^10–12^. Unfortunately, although these types of programs have been reported to be effective, there are few published papers on long-term interventions to date^10,13^, partially because PWS patients are considered quite resistant to interventions aimed at weight loss^14^ and a relatively small percentage of these patients succeed in obtaining benefits from these interventions. It is consequently important to understand the possible predictive factors of responsiveness (or non) to long-term body weight (BW) loss programs.

The aim of this study was to measure the effectiveness of a six-year multidisciplinary metabolic rehabilitation program in adults with PWS and to detect the possible predictive baseline factors determining the long-term BW reduction responsiveness (or not). The longitudinal BW reduction program adopted in the present study included a 3-week hospitalization at baseline, 3 and 6 years of follow-up. Between the three hospitalization periods, a caring team followed up periodically the patients and their families to monitor the clinical conditions and concomitant treatments and to promote positive lifestyle changes.

## Results

Sixty PWS patients, hospitalized at the Division of Auxology, Istituto Auxologico Italiano, Piancavallo (VB), Italy, between December 2003 and January 2020 met the criteria for the current study.

The sample consisted of 38 females and 22 males. Mean age at the time of first admission (T0) was 27 ± 7 y [min–max: 17–46 y], BW was 97 kg ± 29 kg [37–184 kg], height was 1.53 ± 0.09 m [1.33–1.73 m], BMI was 42 ± 12 kg/m^2^, [19–80 kg/m^2^]. After three years (T3) mean BW was 94 ± 24 kg [37–144 kg], BMI was 40 ± 11 kg/m^2^ [19–63 kg/m^2^]. After 6 years (T6) mean BW was 94 ± 23 kg [38–141 kg] and BMI was 40 ± 10 kg/m^2^ [20–60 kg/m^2^].

The patients were divided into 3 subgroups based on their response to the long-term multidisciplinary metabolic rehabilitation program. They were classified responsive if, at T6, they had lost more than 5% of their BW compared to T0, stable if they had maintained BW (± 5%) and non-responsive if they had gained more than 5% of their baseline BW. Twenty-four out of 60 patients (40% of the total) were considered responsive; 17 patients (28%) were classified as stable and 19 patients (32%) were classified as non-responsive. Values of height, BW, BMI and circumferences during the follow-up period are reported in Table 1.

**Table 1:** Age and anthropometric measurements of Prader–Willi syndrome (PWS) patients at baseline, after 3 and 6 years follow-up.

		T0 (no. 60)		T3 (no. 60)		T6 (no. 60)		ΔT3-T0		ΔT6-T0	
		Mean	(SD)	Mean	(SD)	Mean	(SD)	diff	(95%CI)	diff	(95%CI)
Age (y)	R	28	(6)	31	(6)	34	(6)	3^#^	(3 to 3)	6^#^	(6 to 6)
	S	29	(9)	32	(9)	34	(9)	3^#^	(3 to 3)	5^#^	(6 to 5)
	N	24	(5)	27	(5)	29	(6)	3^#^	(3 to 3)	5^#^	(6 to 5)
Height (m)	R	1.54	(0.06)	1.54	(0.06)	1.54	(0.06)	0	(0 to 0)	0	(0 to 0)
	S	1.48*	(0.07)	1.48*	(0.07)	1.48*	(0.07)	0	(0 to 0)	0	(0 to 0)
	N	1.55	(0.09)	1.56	(0.09)	1.56	(0.09)	0	(0 to 0)	0	(0 to 0)
Body weight (kg)	R	110	(28)	99	(24)	94	(20)	− 11^#^	(− 6 to − 16)	− 16^#^	(− 8 to − 24)
	S	90	(26)	91	(28)	91	(25)	1	(5 to − 4)	0	(2 to − 1)
	N	86*	(25)	91	(27)	98	(25)	5^#^	(10 to − 1)	12^#^	(17 to 8)
BMI (kg/m^2^)	R	47	(12)	42	(10)	40	(8.)	− 5^#^	(− 3 to − 7)	− 10^#^	(− 4 to − 10)
	S	41	(11)	41	(11)	41	(10)	0	(2 to − 2)	− 1	(1 to − 1)
	N	36*	(9)	38	(1)	41	(1)	2^#^	(4 to 0)	3^#^	(7 to 3)
Waist circumference (cm)	R	123	(19)	114	(19)	116	(18)	− 9^#^	(− 3 to − 15)	− 7^#^	(− 1 to − 13)
	S	117	(26)	112	(19)	117	(17)	− 5	(10 to − 20)	0	(15 to − 14)
	N	106*	(19)	110	(19)	121	(23)	4	(12 to − 4)	5^#^	(22 to 9)
Hip circumference (cm)	R	136	(17)	125	(14)	124	(12)	− 10^#^	(− 8 to − 13)	− 12^#^	(− 6 to − 18)
	S	125	(22)	120	(19)	127	(16)	− 5	(2 to − 12)	1.5	(7 to 4)
	N	118*	(16)	118	(14)	127	(18)	0	(5 to − 4)	9^#^	(15 to 4)
Waist Hip ratio	R	0.91	(0.09)	0.91	(0.09)	0.95	(0.10)	0.00	(0.04 to − 0.04)	0.04	(0.09 to 0.00)
	S	0.93	(0.11)	0.93	(0.08)	0.92	(0.07)	0.00	(0.11 to − 0.10)	0.00	(0.07to − 0.08)
	N	0.89	(0.08)	0.93	(0.11)	0.95	(0.10)	0.04	(0.09 to − 0.02)	0.06^#^	(0.09 to 0.02)

Measurements are reported as mean and standard deviation (SD), while changes are reported as mean difference and 95% confidence interval (CI). *: significantly different from the responsive subgroup (*p* < 0.05); ^#^: significantly changed compared to baseline. R: responsive patients; S: Stable patients; N: non-responsive patients; BMI: body mass index.

At baseline, non-responsive patients had a lower BW, BMI, waist and hip circumferences than responsive (− 22%, *p* = 0.016; − 24%, *p* = 0.007; − 14%, *p* = 0.020 and − 13% *p* = 0.004) and similar to those stable (*p* = 0.092, *p* = 0.043, *p* = 0.699 and *p* = 0.999). Waist / hip ratio was not significantly different between the three subgroups (*p* = 0.935). Baseline age was similar between subgroups (*p* = 0.073). As far as height is concerned, the stable subgroup showed lower stature than that of the responsive one (− 4%, *p* = 0.048) and similar to the non-responsive subgroup (*p* = 0.051).

As expected by definition, the responsive subgroup significantly decreased BW and BMI at T3 (− 10% and − 11%, *p* < 0.001) and T6 (− 17% and − 18%, *p* < 0.001), while the non-responsive significantly increased their BW and BMI at T3 (+ 6% and + 6%, *p* < 0.001) and T6 (+ 13% and + 13%, *p* < 0.001). Waist and hip circumference also decreased at T3 (− 8% and − 8%, *p* < 0.001) and T6 (− 6% and − 10%, *p* < 0.001) in the responsive subgroup, while they increased in the non-responsive one only at T6 (+ 13%, + 7%, *p* < 0.001). The stable subgroup did not change any of these parameters during the follow-up period. Finally, the waist-to-hip ratio did not change significantly in the three subgroups.

Data of REE, fat-free mass (FFM) and fat mass (FM) are reported in Table 2. At T0, baseline REE, both in absolute terms and relative to FFM, was not significantly different between the three subgroups. FFM in kg and as a percentage of total BW were not significantly different between the three subgroups, while FM in kg was lower in the non-responsive than in the responsive subgroup (− 3%, *p* = 0.013). At T3 and T6 REE decreased only in the responsive subgroup (− 8%, *p* < 0.001 and − 15%, *p* < 0.001), while it did not change significantly in the stable and non-responsive subgroups. REE normalized for FFM (kg) increased only at T6 (+ 11%, *p* = 0.006 in the responsive subgroup, while it remained stable at T3 (*p* = 0.058). No significant changes of REE normalized for FFM (kg) were found in the non-responsive and stable subgroups either at T3 and at T6.

**Table 2:** Resting energy expenditure and body composition of Prader–Willi syndrome (PWS) patients at baseline, after 3 and 6 years follow-up.

		T0 (no. 60)		T3 (no. 60)		T6 (no. 60)		ΔT3-T0		ΔT6-T0	
		Mean	(SD)	Mean	(SD)	Mean	(SD)	diff	(95%CI)	diff	(95%CI)
REE (kcal/die)	R	1734	(405)	1601	(303)	1514	(307)	− 133^#^	(− 3 to − 263)	− 221^#^	(− 92 to − 349)
	S	1556	(347)	1567	(356)	1511	(388)	10	(121 to − 101)	− 45	(96 to − 186)
	N	1543	(331)	1582	(313)	1676	(365)	39	(203 to − 125)	133	(312 to − 46)
REE (kcal/day/kg FFM)	R	40	(7)	37	(5)	36	(6)	− 2^#^	(0.2 to − 5)	− 4^#^	(− 1 to − 8)
	S	41	(7)	41	(7)	40	(10)	0	(4 to − 4)	− 2	(3 to − 6)
	N	40	(8)	41	(5)	41*	(6)	1	(5 to − 4)	− 1	(6 to − 4)
FFM (kg)	R	43.5	(9.2)	43.0	(8.8)	42.6	(7.8)	− 0.4	(1.9 to − 2.7)	− 0.9	(2.0 to − 4.0)
	S	37.8	(8.4)	38.6	(9.4)	38.1	(7.2)	0.9	(4.3 to − 2.6)	0.3	(2.7 to − 2)
	N	38.6	(7.6)	38.6	(6.4)	40.8	(8.6)	0.0	(1.8 to − 1.8)	2.2^#^	(4.2 to 0.2)
FFM (%)	R	40.5	(6.4)	44.5	(8.0)	46.2	(7.3)	4.0	(6.4 to 1.5)	5.7^#^	(8 to 3.4)
	S	43.3	(7.2)	43.9	(6.7)	43.8	(7.9)	0.6	(2.5 to − 1.4)	0.5	(3 to − 2)
	N	46.9	(9.7)	44.8	(11.)	42.8	(8.9)	− 2.1	(0.9 to − 5.01)	− 4^#^	(− 0.6 to − 7.5)
FM (kg)	R	66.4	(21.7)	56.2	(18.6)	51.5	(15.6)	− 10.2^#^	(− 5.5 to − 14.8)	− 15.0^#^	(− 8.4to − 21.3)
	S	52.5	(19.9)	52.4	(20.5)	52.5	(19.1)	− 0.1	(2.1 to − 2.4)	− 0.1	(2.7 to − 2.9)
	N	47.1*	(20.1)	52.4	(24.1)	57.2	(19.6)	5.3^#^	(10.1 to 0.5)	10.1^#^	(14.9 to 5.3)
FM (%)	R	59.5	(6.4)	55.5	(8.0)	53.8	(7.3)	− 4.0^#^	(− 1.5 to − 6.4)	− 5.7^#^	(− 3.4 to − 8)
	S	56.7	(7.2)	56.1	(6.7)	56.2	(7.9)	− 0.6	(1.4 to − 2.5)	− 0.3	(2 to − 3)
	N	53.1	(9.7)	55.2	(11.0)	57.2	(8.9)	2.1	(5.01 to − 0.9)	4^#^	(7.5 to 0.6)

Measurements are reported as mean and standard deviation (SD), while changes are reported as mean difference and 95% confidence interval (CI). *: significantly different from the responsive subgroup (*p* < 0.05); ^#^: significantly changed compared to baseline. R: responsive patients; S: Stable patients; N: non-responsive patients; REE: resting energy expenditure; FFM: fat-free mass; FM: fat mass.

FFM (kg) increased only at T6 in the non-responsive subgroup (+ 5%, *p* = 0.015), while it remained constant in the responsive and stable subgroups at T3 and at T6. Moreover, FM (kg) decreased at T3 (− 18%, *p* < 0.001) and T6 (− 29%, *p* < 001) in the responsive and, conversely, increased in the non-responsive subgroup at T6 (+ 18%, *p* < 0.001).

As far as blood analysis and blood pressure are concerned (Table 3), no significant differences were found between the three subgroups in any of the parameters at any points of the study, apart from glycaemia which was higher at T6 in the stable subgroup compared to the responsive one (+ 40%, *p* = 0.035).

**Table 3:** Biochemical parameters and arterial pressure of Prader–Willi syndrome (PWS) patients at baseline, after 3 and 6 years follow-up.

		T0 (no. 60)		T3 (no. 60)		T6 (no. 60)		ΔT0-T3		ΔT0-T6	
		Mean	(SD)	Mean	(SD)	Mean	(SD)	diff	(95%CI)	diff	(95%CI)
Glycemia (mg/dl)	R	86	(16)	84	(13)	85	(11)	− 2	(3 to − 7)	− 1	(3 to − 6)
	S	94	(19)	103	(30)	121*	(52)	9	(25 − 7)	27	(56 − 3)
	N	93	(26)	97	(34)	94	(20)	3	(10 to − 4)	1	(15 to − 14)
Cholesterol (g/l)	R	181	(29)	186	(36)	181	(34)	5	(19 to − 9)	− 1	(10 to − 11)
	S	178	(40)	195	(45)	180	(28)	17	(58 to − 24)	1	(27 to − 23)
	N	189	(33)	183	(30)	184	(32)	− 6	(9 to − 21)	− 5	(13 to − 23)
HDL (mg/dl)	R	51	(12)	52	(12)	52	(14)	1	(6 to − 5)	1	(6 to − 4)
	S	52	(15)	50	(13)	55	(14)	− 2	(5 to − 9)	2	(9 to − 4)
	N	51	(14)	48	(15)	48	(11)	− 3	(3 to − 8)	− 3	(3 to − 9)
Triglycerides (mg/dl)	R	95	(32)	93	(35)	101	(42)	− 2	(11 to − 14)	6	(21 to − 10)
	S	97	(45)	107	(67)	110	(55)	10	(33 to − 14)	13	(42 to − 17)
	N	105	(41)	96	(41)	101	(35)	− 9	(10 to − 28)	− 4	(17 to − 26)
LDL (mg/dl)	R	119	(25)	120	(31)	115	(31)	1	(13 to − 12)	− 4	(8 to − 16)
	S	111	(32)	117	(36)	113	(24)	6	(22 to − 10)	2	(24 to − 20)
	N	121	(36)	122	(26)	125	(32)	1	(19 to − 17)	4	(22 to − 14)
HbA1c (%)	R	5.6	(0.4)	5.5	(0.5)	5.5	(0.6)	− 0.1	(0.1 to − 0.3)	− 0.2	(0.1 to − 0.5)
	S	6.0	(0.7)	6.1	(1.2)	6.4	(1.9)	0.2	(0.8 to − 0.5)	0.4	(1.6 to − 0.8)
	N	5.9	(1)	5.8	(1.1)	5.7	(0.7)	− 0.1	(0.4 to − 0.5)	− 0.2	(0.3 to − 0.6)
SAP (mmHg)	R	129	(19)	121	(12)	122	(10)	− 8	(4 to − 19)	− 7	(4 to − 19)
	S	121	(14)	122	(12)	122	(6)	1	(12 to − 11)	1	(10 to − 8)
	N	123	(8)	126	(9)	130	(1)	3	(10 to − 4)	7	(15 to − 1)
DAP (mmHg)	R	81	(9)	78	(10)	78	(8)	− 3	(3 to − 9)	− 3	(2 to − 8)
	S	78	(7)	82	(10)	78	(5)	4	(12 to − 4)	0	(6 to − 6)
	N	80	(8)	79	(7)	78	(6)	− 1	(4 to − 6)	− 2	(3 to − 8)

Measurements are reported as mean and standard deviation (SD), while changes are reported as mean difference and 95% confidence interval (CI). *: significantly different from the responsive subgroup (*p* < 0.05); N: non-responsive patients; HDL: high density lipoprotein; LDL: low density lipoprotein; HbA1c: glycated haemoglobin; SAP: systolic arterial pressure; DAP: diastolic arterial pressure.

Finally, there was no different distribution among the three subgroups at T0 of males and females patients treated with hypoglycaemic drugs, GH therapy and smokers (Table 4). Similarly, no different distribution of sex steroid and thyroxine replacement therapy was found in the three subgroups at T0 (data not shown). Conversely, patients with karyotype del15 were more frequent among responsive than non-responsive and stable subgroups; furthermore, patients taking antihypertensive therapy at T0 were more frequent in the responsive than in the other two subgroups (Table 4).

**Table 4:** Frequency of Prader–Willi syndrome (PWS) patients’ main characteristics in responsive, stable and non-responsive subjects at baseline.

		Responsive	Stable	Non-responsive	Chi square
		24 (40%)	17 (28%)	19 (32%)	
Sex	M	9 (38%)	5 (29%)	8 (42%)	0.728
	F	15 (63%)	12 (71%)	11 (58%)	
Karyotype	del15	21 (88%)	12 (71%)	11 (58%)	0.036
	met +	2 (8%)	1 (6%)	0 (0%)	
	UPD	1 (4%)	4 (24%)	8 (42%)	
Diabetes	Yes	2 (8%)	3 (18%)	2 (11%)	0.646
	No	22 (92%)	14 (82%)	17 (89%)	
Glycaemic therapy	Yes	3 (13%)	5 (29%)	4 (21%)	0.407
	No	21 (88%)	12 (71%)	15 (79%)	
Dyslipidaemia therapy	Yes	0 (0%)	0 (0%)	1 (5%)	–
	No	24 (100%)	17 (100%)	18 (95%)	
Antihypertensive therapy	Yes	7 (29%)	2 (12%)	0 (0%)	0.026
	No	17 (71%)	15 (88%)	19 (100%)	
Metabolic Syndrome	Yes	11 (46%)	7 (41%)	6 (32%)	0.634
	No	13 (54%)	10 (59%)	13 (68%)	
Smoke	Yes	4 (17%)	1 (6%)	1 (5%)	0.435
	No	20 (83%)	16 (94%)	15 (79%)	
GH therapy	Yes	9 (38%)	2 (12%)	7 (37%)	0.152
	No	15 (63%)	15 (88%)	12 (63%)	

Variables are reported as the number and proportion of subjects with the characteristic of interest.

*p* < 0.05: frequency significantly different between groups.

Del15: deletion in chromosome 15 Karyotype; Met+ : diagnosis of Prader–Willi through methylation test (Karyotype unknown); UPD: uniparental disomy Karyotype .

## Discussion

This study aimed to report the long-term results of a multidisciplinary metabolic rehabilitation program on PWS patients. First of all the data reported in the present study have several strengths: they are based on 60 patients, who are a large number considering the rarity of PWS, and they are based on a very long-term follow-up period (6 years). Moreover, the recruitment of PWS subjects was made in a single center and their examination was carried out by the same physicians. The multidisciplinary metabolic rehabilitation program was successful in 40% of the patients after 6 years follow-up, while it determined a substantial maintenance (± 5%) of the baseline BW in 28%, which can be considered a “partial” success” taking into account the almost unavoidable progressive increase of BW in these “resistant” patients. In our study population, only 32% gained BW over the 6 years of the study. The interpretation of these findings, however, must take into account that the weight reduction of some patients could be explained, at least in part, by their transition to nutritional phase 4, as described by Miller et al.^15^.

It is interesting to note that the responsive subgroup, who lost BW, did not lose FFM, while the non-responsive subgroup gained an average of 2.2 kg of FFM, this finding being a great success considering the typically worsening fate for this type of population.

These data are important also because very few other published studies are available on the effectiveness of rehabilitation programs in patients with PWS to date^11,16–20^. In particular, two studies have proven that it is possible to maintain or even improve body composition in PWS patients with a multidisciplinary intervention with a follow-up of 13 months^19^ and up to 6 years^17^. The study by Vismara et al. showed that 6-month training program in PWS patients was effective in improving their overall physical functioning, which is very important in this population due to their hypotonia and reduced spontaneous physical activity^16^. Two other publications, evaluating the effects of appropriate energy restriction only, showed the efficacy at 8 months^20^ and at 5–7 years^18^. The large majority of studies were focused on BW loss on average, for example Bedogni et al. reported a decrease of about 5% in BW^11^, similar values being also reported by two other studies^17,19^.

However, not all PWS patients respond in the same way to the long-term multidisciplinary intervention, so focusing on the average result does not help researchers and caregivers in understanding why some respond well to the intervention and others do not. The present study therefore was aimed to analyse the baseline characteristics of responsive, stable and non-responsive subgroups in order to give elements to the scientific community to increase the degree of success of BW reduction programs in PWS patients. Interestingly, the responsive subgroup had baseline higher BMI and waist and hip circumferences than the non-responsive one, however after 6 years all values were similar between subgroups. On the other hand, there were no significant differences at baseline with regard to the measured markers of cardiovascular risk, to the distribution between the subgroups of patients with metabolic syndrome, T2DM or hypertension. There was, however, a difference in the distribution of patients undergoing anti-hypertensive therapy among the responsive subgroup vs. the non-responsive one, probably as a consequence of the fact the former had higher baseline BW and BMI. It should be noted that all metabolic parameters and blood pressure values were similar in all subgroups during the study, with the exception of glycemia in the stable subgroup, and that most of them were within the normal range at all time points of the follow-up, regardless of the response to the program. These findings could be important from a rehabilitation point of view, indicating that long-term multidisciplinary care can favourably control comorbidities in all individuals with PWS^14^.

Finally, another interesting result of the present study is that patients with UPD karyotype were more frequent among the non-responsive and stable subgroups than the responsive one. This could mean that a UPD patient involved in a long-term multidisciplinary BW program has lower probability to respond positively, thus requesting a more careful tailoring of the program itself. To the best of our knowledge this resistance to lose weight related to a specific genetic form has been not previously demonstrated, even if differences in mortality have been previously observed^5,21^. The study of Proffitt et al.^5^ highlighted that individuals with UPD had an increased risk of death from cardiopulmonary factors when compared to del15 patients. Although also the study of Smith et al.^21^ found that UPDs had higher mortality than del15, this finding being however not associated with differences in BW between the two genetic subgroups. In addition to differences in mortality between UPD and del15 individuals, UPD has been reported to be statistically more prone to psychotic-type illnesses and autism^5,7,22^, these concomitant disturbances making them more difficult to be involved in these type of long-term programs in which one of the main purposes is to improve lifestyle.

The present study has some limitations to be taken into consideration. Firstly, the psychiatric trajectory of PWS patients can significantly influence their adherence to long-term rehabilitation programs. Unfortunately, a detailed analysis of the psychological patterns of responder or not-responder patients was not considered in the present study. This aspect will deserve future dedicated investigation. Another weakness of our report is the lack of detailed data on the socio-economic status of the families, which can greatly contribute to successful weight loss. However, all families had at least one member with a paid job. Furthermore, all families received financial support from the Italian social security system for the condition of disability of their son/daughter. For these reasons, it seems unlikely that such factors may significantly contribute in differentiating responses to weight loss. Another limitation is the lack of detailed information on diet and physical activity outside of the hospitalizations collected with the use of specific diaries during the 6-yr follow-up period. Nevertheless, all the patients received specific recommendations about the amount of the caloric intake and adapted physical activity at any clinical control outside of the hospitalization.

In conclusion, 40% of PWS patients showed clinically relevant BW loss at 6 years of follow-up of a multidisciplinary metabolic rehabilitation program, 28% remained stable and 32% gained BW.

Long-term (6 years) responsive PWS patients were characterised by a higher baseline BMI, waist and hip circumferences and del15 genetic pattern, no other predictive factors (body composition, REE, metabolic parameters) considered in the present study contributing to identify the three subgroups. Further future additional studies are mandatory to adapt quickly the BW reduction programs to the individual characteristics of the single PWS patient in a better way.

## Patients and methods

### Subjects

Sixty patients with PWS were selected for a retrospective cohort study. All of them lived at home with their parents. Families were advised to put locks on their kitchens, food cabinets, and refrigerators. Work and social environments have been warned to prevent free access to any food. The patients underwent a long-term multidisciplinary metabolic rehabilitation program at the Division of Auxology, Istituto Auxologico Italiano (Piancavallo, Verbania, Italy).

The patients included in the present study had a genetically confirmed diagnosis of PWS, were older than 17 years and, for each of them, anthropometric data (BW, height, waist and hip circumference and body composition), laboratory data (glucose, triglycerides, cholesterol, high-density lipoprotein (HDL) cholesterol, and low-density lipoprotein (LDL) cholesterol), clinical data (systolic and diastolic blood pressure), and indirect calorimetry data (resting energy expenditure, REE) were available at baseline and at 3 (± 0.5) and 6 (± 0.5) year of follow-up. The study was approved by the Ethics committee of the Istituto Auxologico Italiano (research project code: 01C123, acronym: Mebascocopws) and was conducted in accordance with the Declaration of Helsinki. Written informed consent to participate in the study were provided by all patients and/or their parents or legal guardians, if applicable.

### Anthropometry

Physical examination included determination of height, BW, waist and hip circumference, and waist to hip ratio. BW and height were measured following international guidelines^23^. BMI was calculated as BW (kg)/height (m)^2^ and classified according to the guidelines of the World Health Organization)^24^.

Waist circumference was measured at the midpoint between the last rib and the iliac crest using an anthropometric tape. Hip circumference was measured at the level of anterior superior iliac spine, where this could be felt, otherwise at the broadest circumference below the waist.

### Laboratory and clinical measurements

Haematological parameters such as glucose, glycated haemoglobin, triglycerides, total cholesterol, HDL cholesterol, and LDL cholesterol were measured by our internal laboratory using standardized methods. Following the international guidelines, blood pressure was measured using a sphygmomanometer with appropriately sized cuff, after the participants had been sitting at least 5 min, and the average of three measurements was used for statistical analysis. Metabolic syndrome (MS) was diagnosed using the criteria of the International Diabetes Federation (IDF)^25^.

### Indirect calorimetry

REE was measured between 8:00 and 10:00 a.m. in thermoneutral conditions using a Sensor Medics Vmax 29 (Yorba Linda, CA, USA) metabolic chart equipped with a canopy, as described in detail elsewhere^26^. The patients were in a fasting state for at least 8 h, refrained from physical activity for at least 24 h, and waited at least 30 min in a sitting position before measurement. REE was measured for at least 30 min with patients in a supine position, including an acclimation period of 10 min. The data relative to the acclimation period were discarded. REE was calculated from O_2_ consumption and CO_2_ production using the Weir equation^27^.

### Dual-energy X-ray absorptiometry

Body composition was measured by dual-energy X-ray absorptiometry (DXA) (GE-Lunar Prodigy, GE Medical Systems, Milwaukee, WI, USA). For a detailed description see elsewhere^28^. Percent total [FM (%)] was calculated as (FM (kg)/ BW measured by DXA (kg)) × 100 and segmental FM (%) as (FM legs (kg)/FM (kg)) × 100, (FM arms (kg)/FM (kg)) × 100, and (FM trunk (kg)/FM (kg)) × 100. Fat-free mass (FFM) was obtained by subtracting FM from BW, and percent total FFM as (FFM (kg)/ BW by DXA (kg)) × 100.

### Multidisciplinary metabolic rehabilitation program

The PWS patients underwent a 3-week in-hospital multidisciplinary metabolic rehabilitation program at baseline and at 3 and 6 years of follow-up. The metabolic rehabilitation program followed by obese patients with PWS was generally the same prescribed for non-syndromic obese patients without PWS^29^, only small arrangements being made for specific individual needs. Measurements were performed before the start of each in-hospital period. During the 6-year study period, the patients were periodically followed up both as in-patients (day-hospital) and out-patients by healthcare personnel with qualified expertise in the clinical management of obesity (e.g., endocrinologist, nutritionist, dietician, psychologist, etc.). The three in-hospital periods (T0-T3-T6 yrs) consisted of three weeks of metabolic rehabilitation, entailing energy-restricted diet in combination with physical rehabilitation (moderate aerobic activity) and nutritional information. A Mediterranean diet was prescribed in all cases, and the amount of energy to be given with diet was calculated by subtracting approximately 500 kcal from the measurement of REE. The physical activity program consisted of 5 days of training per week, including: (i) 1 h of supervised moderate whole body aerobic exercise (arms and legs together), (ii) either 20–30 min of cycloergometer exercise at 60 W, or 3–4 km out-door walking on flat terrain. At the end of third week of the rehabilitation program, the patients and their caregivers received individualized counselling on nutrition and physical activity^16^. A multidisciplinary team promoted lifestyle changes for the entire duration of the study.

### Statistical analysis

Variables are reported as mean and standard deviation, while changes outcomes of interest are reported with 95% confidence interval (95% CI). Statistical analyses were performed using GraphPad Prism 8.0 with significance set at *p* < 0.05. Normal distribution of the data was tested using the Shapiro–Wilk test. Analyses of repeated measures data (time at T0, T3, T6), between factor (groups) and interactions (time x group) were carried out with a mixed-effects model using the restricted maximum likelihood method. When significant differences were found, a Bonferroni post hoc test was evaluated implementing multiple comparison. Furthermore, categorical variables’ prevalence within the three groups were analysed through chi-square test.

## Data Availability

The datasets generated during the current study are available from the corresponding author upon a reasonable request.

## References

[1] Bar C (2017). Early diagnosis and care is achieved but should be improved in infants with Prader–Willi syndrome. Orphanet. J. Rare Dis..

[2] Angulo MA, Butler MG, Cataletto ME (2015). Prader–Willi syndrome: A review of clinical, genetic, and endocrine findings. J. Endocrinol. Invest..

[3] Butler MG (2019). Molecular genetic classification in Prader–Willi syndrome: A multisite cohort study. J. Med. Genet..

[4] Miller JL (2011). Nutritional phases in Prader–Willi syndrome. Am. J. Med. Genet. A.

[5] Proffitt J (2019). Contributing factors of mortality in Prader–Willi syndrome. Am. J. Med. Genet. A.

[6] Reus L (2011). Motor problems in Prader–Willi syndrome: A systematic review on body composition and neuromuscular functioning. Neurosci. Biobehav. Rev..

[7] Cassidy SB, Schwartz S, Miller JL, Driscoll DJ (2012). Prader–Willi syndrome. Genet. Med..

[8] Butler MG, Theodoro MF, Bittel DC, Donnelly JE (2007). Energy expenditure and physical activity in Prader–Willi syndrome: Comparison with obese subjects. Am. J. Med. Genet. A.

[9] Bellicha A (2021). Physical activity in patients with Prader–Willi syndrome—A Systematic Review of Observational and Interventional Studies. J. Clin. Med..

[10] Duis J (2019). A multidisciplinary approach to the clinical management of Prader–Willi syndrome. Mol. Genet. Genom. Med..

[11] Bedogni G (2020). Changes of body weight and body composition in obese patients with Prader–Willi syndrome at 3 and 6 years of follow-up: A retrospective cohort study. J. Clin. Med..

[12] Hirsch HJ (2021). Long-term weight control in adults with Prader–Willi syndrome living in residential hostels. Am. J. Med. Genet. A.

[13] Morales JS (2019). Physical exercise and Prader–Willi syndrome: A systematic review. Clin. Endocrinol. (Oxf)..

[14] Crinò A, Fintini D, Bocchini S, Grugni G (2018). Obesity management in Prader–Willi syndrome: Current perspectives. Diabetes Metab. Syndr. Obes..

[15] Miller JL (2011). Nutritional phases in Prader–Willi syndrome. Am. J. Med. Genet. A.

[16] Vismara L (2010). Effectiveness of a 6-month home-based training program in Prader–Willi patients. Res. Dev. Disabil..

[17] Grolla E (2011). Specific treatment of Prader–Willi syndrome through cyclical rehabilitation programmes. Disabil. Rehabil..

[18] Miller JL, Lynn CH, Shuster J, Driscoll DJ (2013). A reduced-energy intake, well-balanced diet improves weight control in children with Prader–Willi syndrome: Weight control in children with Prader–Willi syndrome. J. Hum. Nutr. Diet..

[19] Hauber M, Stratmann B, Hoedebeck-Stuntebeck N, Tschoepe D (2013). Medical management for adults with Prader–Willi syndrome. Metab. Syndr. Relat. Disord..

[20] de Lima VP (2016). Nutritional intervention with hypocaloric diet for weight control in children and adolescents with Prader–Willi Syndrome. Eat. Behav..

[21] Smith A (2003). Death in adults with Prader–Willi syndrome may be correlated with maternal uniparental disomy. J. Med. Genet..

[22] Boer, H. *et al.* Psychotic illness in people with Prader Willi syndrome due to chromosome 15 maternal uniparental disomy. *Lancet.* **359**, 135–136. 2 (2002). 10.1016/S0140-6736(02)07340-3 11809260

[23] Pelletiet, D. *Anthropometric Standardization Reference Manual: Abridged* edition (ed. by Lohman, T. G., Roche, A. F. & Martorell, R.) (Human Kinetics Books, Champaign, IL, 1991).

[24] World Health Organization. Obesity and Overweight. WHO Fact Sheet No. 311. 2014. Available online: http://www.who.int/mediacentre/factsheets/fs311/en/

[25] Alberti KGMM (2009). Harmonizing the metabolic syndrome: A joint interim statement of the International Diabetes Federation Task Force on epidemiology and prevention; National Heart, Lung, and Blood Institute; American Heart Association; World Heart Federation; International Atherosclerosis Society; and International Association for the Study of Obesity. Circulation.

[26] Bedogni G (2019). External validation of equations to estimate resting energy expenditure in 14952 adults with overweight and obesity and 1948 adults with normal weight from Italy. Clin Nutr..

[27] de Weir JBV (1949). New methods for calculating metabolic rate with special reference to protein metabolism. J. Physiol..

[28] Bedogni G (2019). Assessment of fat-free mass from bioelectrical impedance analysis in men and women with Prader–Willi syndrome: Cross-sectional study. Int. J. Food Sci. Nutr..

[29] Rigamonti AE (2020). Effects of a 3-week in-hospital body weight reduction program on cardiovascular risk factors, muscle performance, and fatigue: A retrospective study in a population of obese adults with or without metabolic syndrome. Nutrients.

