# Author Correction: Predictive factors of responsiveness to a body weight reduction program in Prader–Willi patients at 6 years of follow‑up

**DOI:** 10.1038/s41598-022-14562-7

**Published:** 2022-06-17

**Authors:** Stefano Lazzer, Filippo Vaccari, Mattia D’Alleva, Giorgio Bedogni, Diana Caroli, Graziano Grugni, Alessandro Sartorio

**Affiliations:** 1 Department of Medicine, University of Udine, P.le Kolbe 4, 33100 Udine, Italy; 2 School of Sport Sciences, University of Udine, 33100 Udine, Italy; 3 Department of Medical and Surgical Sciences, Alma Mater Studiorum, University of Bologna, 40138 Bologna, Italy; 4 Experimental Laboratory for Auxo-Endocrinological Research, Istituto Auxologico Italiano, IRCCS, 28824 Piancavallo, VB Italy; 5 Division of Auxology, Istituto Auxologico Italiano, IRCCS, 28824 Piancavallo, VB Italy

Correction to: *Scientific Reports* https://doi.org/10.1038/s41598-022-09096-x, Published online 25 March 2022

The original version of this Article contained an error in the Funding section.

“This research was funded by Progetti di Ricerca Corrente, Istituto Auxologico Italiano IRCCS, Milan, Italy (research project code: 01C123, acronym: Mebascocopws).”

now reads:

“Research funded by the Italian Ministry of Health.”

The original Article has been corrected.

